# Supplementary material for: Isotopic evidence of acetate turnover in Precambrian continental fracture fluids
Source: Nat Commun. 2024 Oct 23;15:9130. doi: 10.1038/s41467-024-53438-4 (PMC11499883; doi:10.1038/s41467-024-53438-4)
Supplement: Supplementary file 1 — Supplementary Information [file 41467_2024_53438_MOESM1_ESM.pdf]

Supplemental Information for "Isotopic Evidence Of Acetate Turnover In Precambrian Continental Fracture Fluids"

Elliott P. Mueller*[1], Juliann Panehal[1], Alexander Meshoulam[1], Min Song[2], Christian T. Hansen[3], Oliver Warr[2,4], Jason Boettger[5], Verena B. Heuer[3], Wolfgang Bach[3], Kai-Uwe Hinrichs[3], John M. Eiler[1], Victoria Orphan[1], Barbara Sherwood Lollar[2,6], Alex L. Sessions[1]

[1] Division of Geological and Planetary Sciences, California Institute of Technology, Pasadena, CA, USA
[2] Department of Earth Sciences, University of Toronto, Toronto, ON, Canada
[3] MARUM Centre for Marine Environmental Sciences, University of Bremen, Bremen, Germany
[4] Department of Earth Sciences, University of Ottawa, Ottawa, ON K1N 6N5, Canada
[5] Department of Earth, Environmental, and Resource Sciences, University of Texas at El Paso, El Paso, Texas, USA
[6] Institut de Physique du Globe de Paris (IPGP), Université Paris Cité, 1 rue Jussieu, 75005, Paris, France
*Corresponding author: elliottpmueller@gmail.com

Acetate was purified from the high salinity matrix of fracture fluids prior to isotopic analysis. To verify that the purification scheme did not introduce isotopic fractionation, we spiked synthetic solutions of Kidd Creek fluid (Table S1) with acetate of known isotopic composition ($\delta^{13}C$ = -19.2‰, $\delta^{2}H$ = -127‰) and then reanalyzed them after extraction from the solution. Replicates of 1 mM and 2 mM acetate solutions yielded identical $\delta^{13}C$ and $\delta^{2}H$ values within analytical uncertainty, indicating that the extraction procedure is not fractionating. (Figure S1). Small differences <1‰ between the measured and expected values are common in ESI-Orbitrap measurements, even when comparing standards of pure solutions of acetate. This is reported in detail in the original method development study. The source of these minor inaccuracies are still unclear.[1]

Component	Concentration (g/L)
$CaCl_2$	144.274
NaCl	36.817
KCl	0.201
$MgCl_2$	8.378
NaBr	2.469
$NaNO_3$	0.017
$NaNO_2$	0.014
KH_2PO_4	0.027
$MgSO_4$	0.072
Na-formate	0.034

Table S1: Composition of synthetic solution used for validation studies on acetate purification techniques.

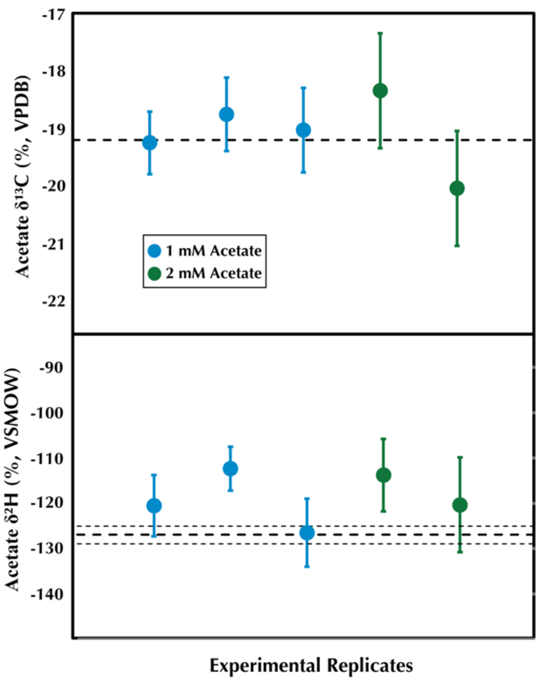

Figure S1: Extracted acetate standard from a synthetic fracture fluid mixture has the same $\delta^{13}C$ and $\delta^{2}H$ values as the known composition. Error bars are one standard deviation on triplicate analytical replicates. Dotted lines represent the reported value of the standard along with uncertainties on $\delta^{2}H$ values. Uncertainties are 0.1‰ on the reported $\delta^{13}C$ composition and are not visible on this plot.

Location	Kidd Creek	Kidd Creek	Kidd Creek	Kidd Creek	Kidd Creek	Kidd Creek	Birch Tree	Birch Tree	Birch Tree
Borehole	12261	12299	12287	12299	12299	12261	1113860	1113740	BH1A
Average $\delta^{13}C$	-8.8	-7.0	-6.6	-8.6	-9.4	-10.0	-26.8	-27.4	-26.7
Standard Deviation $\delta^{13}C$	0.5	0.2	0.8	1.2	0.7	0.9	0.9	0.7	0.6
Average $\delta^{2}H$	-134.1	-129.5	-142.5	-133.6	-134.8	-130.2	-169.8	-166.5	-170.2
Standard Deviation $\delta^{2}H$	3.2	3.8	2.1	3.0	4.9	3.7	5.4	7.1	7.9
Water* $\delta^{2}H$	-36	-36	-36	-36	-36	-36	-74	-74	-74
Alpha (ac/w)	0.898	0.903	0.890	0.899	0.898	0.902	0.897	0.900	0.896
Acetate Conc. (mM) **	1.6	1.4	1.9	1.4	1.4	1.6	0.15	0.23	0.27
Temperature (C) **	25	25	25	25	25	25	21	21	21
Depth **	2.4 km	2.4 km	2.4 km	2.4 km	2.4 km	2.4 km	--	--	--
Sampling Date	28/08/2007	27/08/2007	28/08/2007	27/08/2007	2018	2018	7/11/07	8/11/07	28/05/2007
Method of measurement	Dual Inlet	Dual Inlet	Dual Inlet	Dual Inlet	Dual Inlet	Dual Inlet	Direct Infusion	Direct Infusion	Direct Infusion

Table S2: Acetate isotope composition and metadata from Kidd Creek and Birchtree fluids. *Taken from Warr et al. 2021[2]. **Taken from Sherwood Lollar et al. 2021.[3]

Experiment Date	Temperature (°C)	Half-time (hr)	Half-time (yr)
February 2023	60	7.1×10^6	811
February 2023	60	6.5×10^6	742
June 2022	100	2.7×10^4	3.08
April 2022	150	112	0.012
June 2022	150	187	0.021
January 2023	200	3	0.00034
January 2023	200	3	0.00034

Table S3: Half-times of equilibration determined at temperatures between 60 and 200°C. Data used to make Figure 1 in the main text.

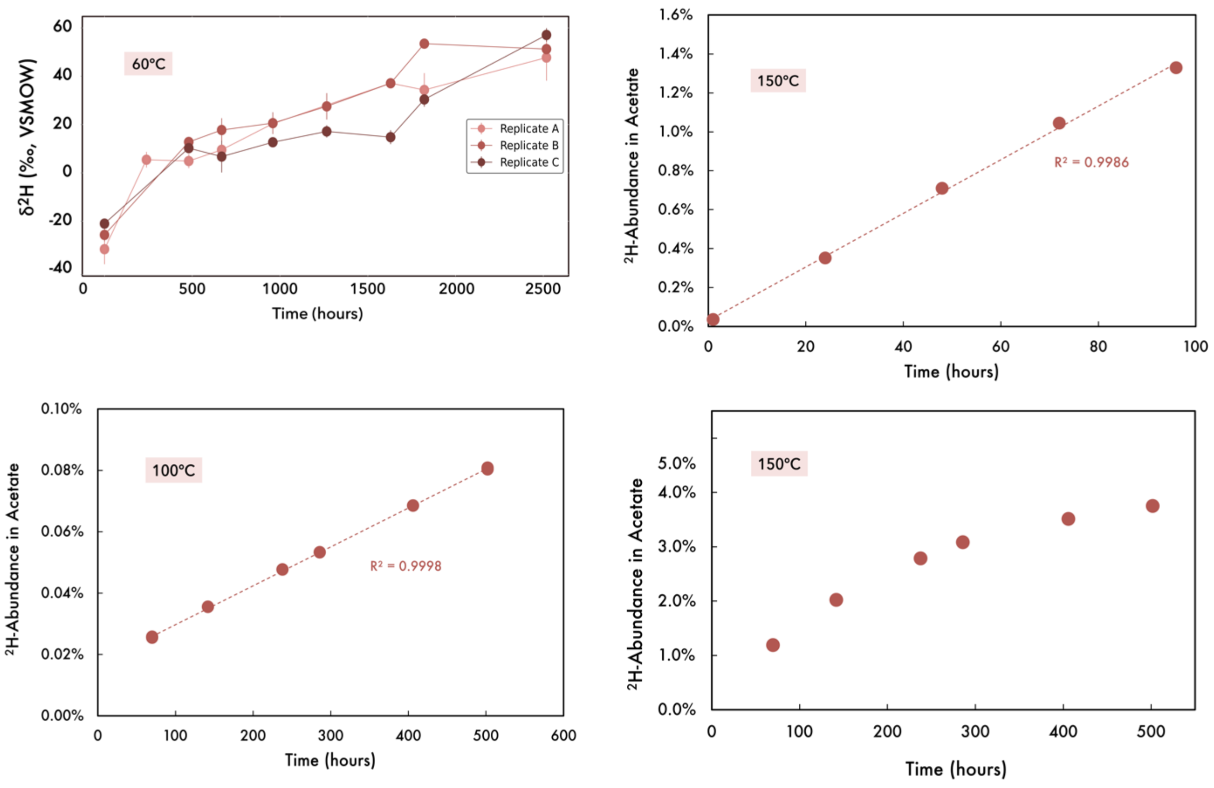

Figure S2: Exchange experiments in deuterated water (5% ^2H_2O) with 1 mM acetate at 60°C, 100°C, and 150°C. Acetate incorporated deuterium from the water into its methyl-site in a linear fashion. At 60°C, the exchange rate is so low that deuterium incorporation is quantified as changes in natural abundance δ^2H values (10‰ is equivalent to 1 ppm absolute increase). All measurements made on an electrospray Orbitrap mass spectrometer. Error bars represent standard deviation of analytical triplicates.

To account for changes in exchange reaction rates due to complexation of acetate with inorganic cations in solution, acetate was incubated in 1 mol/L $CaCl_2$ for 4 hours at 120°C. This experiment was done in triplicate. After the incubation, acetate was enriched by 191‰ ± 4‰. Uncertainty represents standard deviation of experimental replicates, which was equivalent to analytical error. This enrichment with time was converted to a reaction rate of exchange at 120°C, which falls on the established Arrhenius relationship between temperature and reaction rate based on exchange with pure water. These data suggest that complexation with calcium cations, the major cation in Kidd Creek, does not impact exchange kinetics.

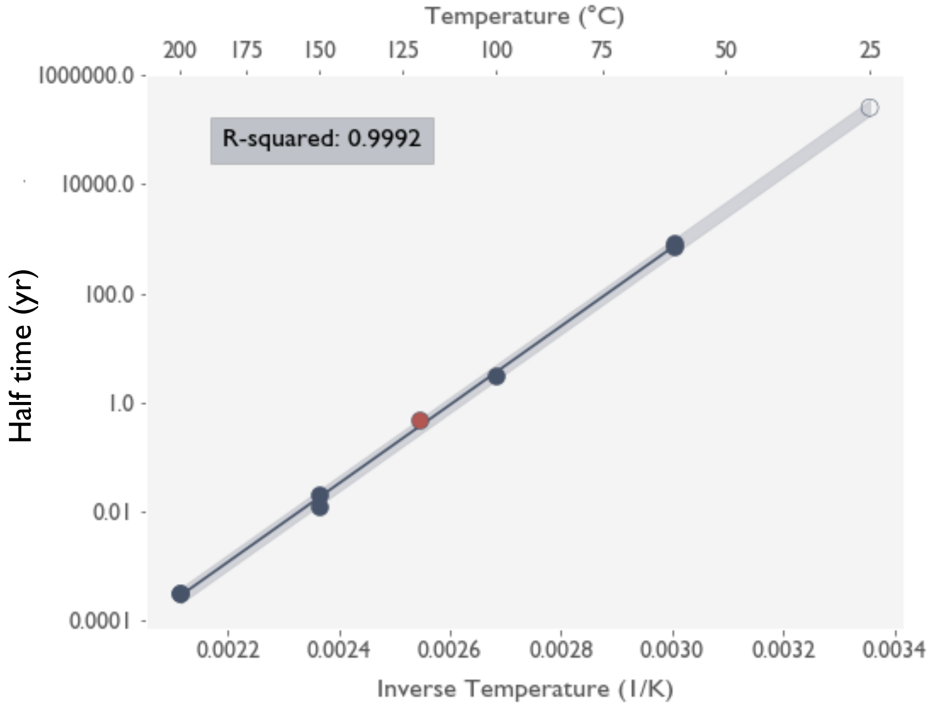

Figure S3: Arrhenius plot of exchange reaction rate with temperature (from Figure 1 in main text) for acetate in deuterated water (5% ^2H_2O). Dark blue dots represent exchange with pure water. Red dot represents exchange in 1M $CaCl_2$ at 120°C.

The tautomerization reaction that putatively exchanges hydrogen atoms with water is shown below (Figure S4). Tautomerization reactions are well-documented isomerizations between carbonyl/carboxyl and enol forms of organic ketones and acids. [4,5] The good agreement with the Arrhenius equation between 60°C and 200°C suggests that the reaction mechanism of exchange does not change in this temperature range. Experiments at lower temperatures are not possible on laboratory timescales due to the long time for exchange (thousands of years). At the *in situ* temperature of Kidd Creek and Birchtree (~25°C), the extrapolated half-time of exchange is 250,000 years. If the relationship between temperature and rate were to deviate from the regression at lower temperatures, it would represent a shift in exchange mechanism. This new mechanism would have to have a faster rate at 25°C than the one measured at high temperatures,

otherwise this mechanism would not be rate limiting. If this new mechanism was slower, the estimated rate of exchange by tautomerization would not be impacted, as it would still be rate limiting. This means that the exchange rate is a conservative, minimum estimate. If another mechanism was rate-limiting, our extrapolation would be an overestimate of the exchange half-time in Kidd Creek and Birchtree, and faster exchange rates only further the conclusions drawn in the following sections regarding acetate cycling rates.

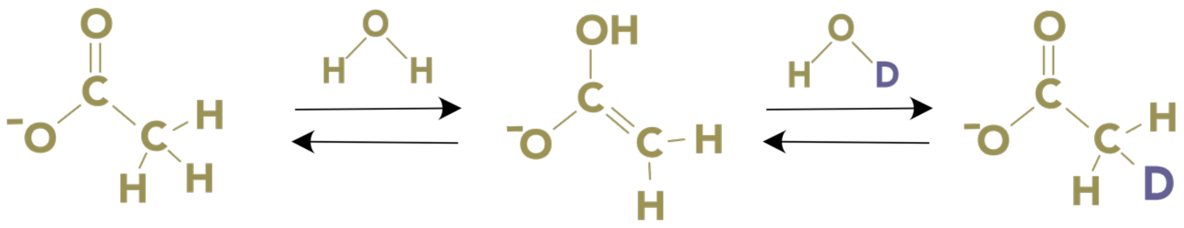

Figure S4: Proposed tautomerization reaction mechanism for exchanging hydrogen isotopes between water and the methyl group of acetate.

DFT Calculations of Water: Continuum solvation models can fail to accurately reproduce isotopic effects of H involved in hydrogen interactions (e.g. H_2O molecule dissolved in water) and therefore a special treatment is required.[6] To overcome this problem we derived the EIE value of H_2O in liquid water using the experimental alpha liquid/vapor and the $^2H/^1H$ ratio in H_2O calculated in the gas phase. Using this approach, we were able to obtain $^2\varepsilon_{(acetate/water)}$ values which are similar to the experimental values (Fig. 2A).

Complexation of calcium and acetate: Influences on the equilibrium isotope effect.

In $CaCl_2$ brines, like the Kidd Creek and Birchtree fluids, calcium cations and acetate anions can complex to form bidentate structures. If this is a major form of acetate in the fracture fluids, then the acetate/water equilibrium isotope effect (EIE) must be determined for both the free acetate ion and the Ca-acetate complex. In the main text, the equilibrium isotope effect is calculated in three different ways: 1.) Between free acetate and pure water, 2.) between free acetate and a 3M $CaCl_2$ brine and 3.) between a calcium-acetate complex and a 3M $CaCl_2$ brine. The first two calculations (free acetate-water equilibrium and free acetate-brine equilibrium) were performed as described in the methods section of the main text.

However for the third calculation, a molecular model of the Ca-acetate complex was created. To do so, we started with a molecular geometry of the bidentate complex which was previously determined by Muñoz Noval et al. (2018).[7] Their empirical structure was used as an initial condition to determine the optimized electronic structure of the Ca-acetate complex. The DFT calculations used identical methods as in the main text. An EIE value representing a ^2H substitution at each of the three H atoms on the methyl group was calculated. The average value of the three was used as the overall EIE.

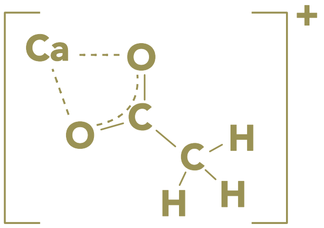

Figure S5: Diagram of the bidentate calcium-acetate complex that can form in high $CaCl_2$ brines, like those of Kidd Creek and Birchtree. Bond lengths and angles are not to scale.

During these calculations, the following optimized atomic coordinates were determined for the free acetate and Ca-acetate complex (Tables S4a and S4b, respectively).

Table S4a. Coordinates of the optimized structure for $C_2H_3O_2^-$

Atom	X	Y	Z
C	-1.351609	-0.039572	-0.002275
H	-1.750804	0.637911	-0.762956
H	-1.735935	-1.04737	-0.173155
H	-1.722086	0.316133	0.96555
C	0.199222	-0.000106	-0.005931
O	0.798162	-1.104908	0.001285
O	0.717231	1.146332	0.00119

Table S4b. Coordinates of the optimized structure for $C_2H_3O_2Ca$

Atom	X	Y	Z
C	-2.471200	-0.000006	0.063380
H	-2.892009	0.895852	-0.392797
H	-2.892007	-0.895864	-0.392795
H	-2.742121	-0.000001	1.123917
C	-0.961595	0.000001	-0.043604
O	-0.340032	-1.106705	-0.069206
O	-0.340037	1.106713	-0.069063
Ca	1.728173	-0.000001	0.032458

Acetate and the Ca-acetate complex had the following calculated frequencies (Tables S5a and S5b)

Table S5a. Calculated frequencies (cm^{-1}) for – $C_2H_3O_2^-$ (acetate in water)

34.3949	436.5932	611.1575
635.6974	884.2003	998.3083
1036.549	1332.708	1378.981
1454.293	1469.448	1651.552
3010.94	3069.027	3092.507

Table S5b. Calculated frequencies for (cm^{-1}) – $C_2H_3O_2Ca^+$		
36.3313	102.7298	200.8444
290.1095	460.3864	614.7865
681.1430	940.0897	1023.8721
1064.3274	1368.8363	1443.1461
1446.9535	1476.3733	1554.2087
3040.1641	3106.1612	3140.4640

The optimized molecular structures of acetate and the Ca-acetate complex had the following geometry (Tables S6a and S6b):

Table S6a. Calculated molecular geometry for– $C_2H_3O_2^-$

C-O-C angle	127.218°
C-O bond length (upper/ lower)	1.258 Å / 1.257 Å
C-C bond length	1.551 Å
Dihedral angle H(lower) -C-C-O (lower)	-10.21093°

Table S6a. Calculated molecular geometry for– $C_2H_3O_2Ca^+$

O-C-O angle	121.319°
C-O bond length upper/ lower	1.2696 Å / 1.26956 Å
O-Ca bond length	2.34789 Å / 2.34790 Å
C-C bond length	1.51339 Å
Dihedral angle H(lower) -C-C-O (lower)	-29.37205°
C-Ca length	2.69084Å

High ionic strength solutions also have lower dielectric constants for water, which would influence the force field that simulates water solvation in the DFT calculations. We tested whether this would change the calculated EIE by solving for the optimized electronic structures of free acetate and the Ca-acetate complex using two different dielectric constants for water: 60 and 40. However, changing the dielectric constant of water did not shift the calculated EIEs by more than 1‰ at 25°C. Thus, our DFT models predict that the change in dielectric constant of water does not influence calculated partition function ratio of acetate or the Ca-acetate complex.

Supplementary References

1. Mueller, E. P., Sessions, A. L., Sauer, P. E., Weiss, G. M. & Eiler, J. M. Simultaneous, High-Precision Measurements of δ ^{2} H and δ ^{13} C in Nanomole Quantities of Acetate Using Electrospray Ionization-Quadrupole-Orbitrap Mass Spectrometry. *Anal. Chem.* **94**, 1092–1100 (2022).
2. Warr, O. *et al.* High-resolution, long-term isotopic and isotopologue variation identifies the sources and sinks of methane in a deep subsurface carbon cycle. *Geochim. Cosmochim. Acta* **294**, 315–334 (2021).
3. Sherwood Lollar, B. *et al.* A window into the abiotic carbon cycle – Acetate and formate in fracture waters in 2.7 billion year-old host rocks of the Canadian Shield. *Geochim. Cosmochim. Acta* **294**, 295–314 (2021).
4. Mardyukov, A., Eckhardt, A. K. & Schreiner, P. R. 1,1-Ethenediol: The Long Elusive Enol of Acetic Acid. *Angew. Chem. Int. Ed.* **59**, 5577–5580 (2020).

5. Ouellette, R. J. & Rawn, J. D. Condensation Reactions of Carbonyl Compounds. in *Organic Chemistry* 747–801 (Elsevier, 2014). doi:10.1016/B978-0-12-800780-8.00022-X.
6. Boettger, J. D. & Kubicki, J. D. Equilibrium and kinetic isotopic fractionation in the CO2 hydration and hydroxylation reactions: Analysis of the role of hydrogen-bonding via quantum mechanical calculations. *Geochim. Cosmochim. Acta* **292**, 37–63 (2021).
7. Muñoz Noval, Á., Nishio, D., Kuruma, T. & Hayakawa, S. Coordination and structure of Ca(II)-acetate complexes in aqueous solution studied by a combination of Raman and XAFS spectroscopies. *J. Mol. Struct.* **1161**, 512–518 (2018).
